# An early evaluation of team consistency and scope optimization in team-based cancer care

**DOI:** 10.1186/s12885-025-13644-9

**Published:** 2025-02-28

**Authors:** Leah K. Lambert, Farinaz Havaei, Scott M. Beck, Andy Ma, John Larmet, Jagbir Kaur, Nassim Adhami, Dan Le, Ryan Woods

**Affiliations:** 1 BC Cancer, Provincial Health Services Authority, Vancouver, Canada; 2 School of Nursing, University of British Columbia, Vancouver, Canada; 3 Faculty of Medicine, University of British Columbia, Vancouver, Canada

**Keywords:** Cancer care, Team-based care, Team composition, Scope of practice, Team consistency, Team effectiveness

## Abstract

**Background:**

The British Columbia (BC) government has made significant investments towards the implementation of team-based care (TBC) in its provincial comprehensive cancer control program. TBC implementation involves purposeful efforts towards: (a) establishing/expanding multidisciplinary care teams, (b) optimizing scope of practice, and (c) increasing care team consistency. Study objectives include an early-phase evaluation of (i) the association between TBC elements and team effectiveness and (ii) staff perceptions of barrier and facilitators of team effectiveness.

**Methods:**

A series of five surveys over a 2-year period will be administered to prospectively evaluate the ongoing implementation of TBC. This study draws on data from the first of the five planned surveys, administered in May 2023. Eligible respondents included 299 program employees—spanning various roles such as physicians, nurses, and unit clerks—working within TBC at the time of survey deployment. The survey included both validated and researcher-developed questions that were either closed or open-ended, including measures of team composition, team consistency, team effectiveness, scope of practice, and demographics. Quantitative data were analyzed using descriptive and regression analysis; qualitative data were analyzed guided by interpretive description methodology.

**Results:**

Collected responses totaled 121, with the majority of respondents being women (76%), full-time employees (90%), and working in direct patient care (77%). Regression analyses indicated that (i) higher frequency of consistently working with the same team members and (ii) lower proportion of shifts practicing below scope are both significant predictors of higher team effectiveness ratings. Qualitative data highlighted staffing levels as a driver of under- and over-utilized scopes of practice. Furthermore, effective communication, enhanced knowledge of each team member’s scope of practice, and strong interpersonal relationships were highlighted as contributing factors to effectiveness among multidisciplinary care teams.

**Conclusions:**

Preliminary findings from the first of five prospective surveys highlight team consistency and role optimization as drivers of effective teamwork in the early implementation of a team-based model of cancer care. Future research should explore contextual factors that influence cancer care staff and clinicians’ perceptions of effectiveness.

## Background

Over recent decades, the cancer care landscape has been reshaped by the rapid advancements and increasing complexity of cancer treatments (e.g., complex immunotherapy, genomic medicine, theranostics, etc.) and the rising incidence of cancer, which has strained conventional models of ambulatory cancer care; therefore, new models of cancer care must be considered [1–3]. One of these approaches is team-based care (TBC), broadly defined as two or more health providers working collaboratively to provide comprehensive, continuous, coordinated, and high-quality care services to patients, families, and communities [4, 5]. In Canada, TBC has gained popularity since its introduction to primary care settings [6]. In 2023, the Government of British Columbia (BC) launched its *10-year Cancer Action Plan* to strengthen the sustainability, interdisciplinarity, accessibility, and coordination of cancer care services at BC Cancer—the publicly-funded cancer control program for the Province of BC—through the provincial implementation of a new model of TBC in its six regional Cancer Centres [7].

This study is part of a larger two-year longitudinal survey study aimed at prospectively evaluating the impact of TBC among oncology staff (i.e., clerical and administrative staff) and clinicians (i.e., physicians, nurse practitioners [NP], registered nurses [RN], licensed practical nurses [LPN], patient care aides [PCA], clerical staff, Indigenous Patient Navigators [IPNs], pharmacists, and other allied health clinicians). The experiences and perspectives of patients who receive TBC at BC Cancer are being considered separately in a parallel body of work. In this manuscript we report baseline findings from data collected during the first timepoint of the study, which was administered during the early phase of TBC implementation at BC Cancer. The overall study explores three research questions: (1) what are oncology staff and clinicians’ perceptions of care delivery (i.e., the provision or coordination of biopsychosocial cancer services) and work experiences (i.e., the perspectives of staff and clinicians who provide or coordinate biopsychosocial cancer services)? (2) what is the association between TBC elements and team effectiveness? and (3) what are the facilitators and barriers of team effectiveness during the initial phase of TBC implementation? The study findings will inform iterative refinements and improvements to the design and implementation of the TBC model in oncology. We posit that aligning TBC more closely with the needs of oncology staff and clinicians will enhance their ability to provide collaborative, high-quality, and patient-centered care, that lead to better experiences, and potentially outcomes, for cancer patients.

The aim of TBC at BC Cancer is threefold: first, TBC aims to support and enable staff and clinicians to optimize their roles and scopes of practice, thus allowing team members to enact the fullest extent of their training and professional competencies. Second, TBC seeks to develop—and later expand—multi-disciplinary, high-functioning, and integrated care teams. Phase 1 teams are comprised of physicians, NPs, RNs, LPNs, clerical staff, and/or IPNs; Subsequent phases will introduce additional allied health clinicians, including clinical pharmacists and radiation therapists. These integrated teams of staff and clinicians will work collaboratively to provide enhanced timely, comprehensive, and person-centered cancer care. Last, TBC aims to improve satisfaction in care among patients and caregivers through a greater focus on longitudinal relationships with an integrated team of staff and clinicians.

## Literature review

This literature review provides conceptual definitions of key study variables and reviews the state of the literature related to the three research questions.

Effective teamwork is characterized by factors including diversity of team composition, clarity of roles and responsibilities, a shared understanding of team goals, trusting relationships, anticipation and response to team members’ needs, and effective communication [8]. In primary care, decentralized models of care, disciplinary hierarchies, and siloed educational systems are among the documented barriers of effective teamwork [9, 10]. It is not clear if these factors are similar or different in cancer care.

Although the implementation of TBC may require different elements and strategies depending on context, this model of care usually involves intentional consideration of the following: team composition, team size, and team members’ scope of practice [3, 8–12]. Compared to traditional models of care, TBC has been positively associated with more effective teamwork [3, 13, 14]. An extensive body of evidence also supports the association between TBC and improved outcomes for patients (e.g., improved clinical and quality outcomes) [3, 13–16, 19], staff/clinicians (e.g., increased job satisfaction, decreased burnout) [13, 14, 19], and organizations (e.g., reduced readmissions and staff overtime) [13, 14, 17–19].

Team composition refers to care team membership, which can consist of members of same or different disciplines [3]. Compared to intradisciplinary care teams, which consists of healthcare workers from the same field or discipline (e.g., a team of nurses), multidisciplinary care teams that bring together professionals from diverse disciplines (e.g., physicians, nurses, social workers) are gaining popularity. A systematic review of 16 international studies found improved cancer treatment planning, medication adherence, and pain control in multidisciplinary teams that leverage the specialized knowledge and expertise of various staff and clinicians [3]. Similar to this systematic review [3], another systematic review of 51 studies that examined TBC in cancer care found that teams with a multidisciplinary composition improved a series of quality outcomes for patients, including clinical diagnostic and treatment decision-making and survival [16].

Scope of practice refers to pre-defined expectations associated with the roles and duties that all members of a discipline are educated and legislated to perform at an entry to practice level [8, 20–22]. Most research on this topic has been conducted with the nursing workforce. Two Canadian survey studies showed that a notable proportion of nurses in rural and remote areas (16% of RNs and 23% of LPNs) practice either below or above their full scope of practice [23, 24]. Research suggests that nurses in urban settings are often not practicing to full scope, meaning that certain nursing functions that could be provided by nurses are instead not being delivered by them [20, 25, 26]. For example, cross-sectional survey studies of 301 and 222 pediatric and maternity nurses in Urban hospitals in Quebec found that on average nurses adhered to a full scope of practice only occasionally or less than frequently in their daily work (scores ranging from 3.2 to 3.7 out of a possible 6-point scale). Cross-sectional qualitative or survey research in various settings has linked an underutilized scope of practice to job dissatisfaction for staff, missed opportunities for better patient care, and higher costs for healthcare organizations [20–25]. In the context of cancer care, a qualitative study of conveniently selected nurses (*n* = 4) and radiation therapists (*n* = 4) in one Cancer Centre found that a lack of understanding of roles and scopes among multidisciplinary care teams contributes to team conflict [27]. Our study further investigates the association between multidisciplinary scopes of practice and team effectiveness.

Care team consistency provides greater opportunities for team members of all disciplines to establish trusting relationships, form a shared understanding, anticipate and respond to each other’s needs, and practice effective communication—all of which are characteristics of high functioning teams [28, 29]. Most of the research on this topic has been conducted in surgical settings where care team consistency in operating rooms was linked to better outcomes for patients (e.g., reduced length of hospital stays and readmission rates) and organizations (e.g., surgical efficacy outcomes) [28, 29]. There is a need to better understand care team consistency in cancer care settings.

The introduction of TBC at BC Cancer presents an opportunity to prospectively evaluate the work and care delivery experiences of oncology staff and clinicians as implementation advances over time. It is widely recognized that stressful working conditions put staff and clinicians at risk of burnout, job dissatisfaction, high turnover, and the potential of suboptimal care delivery [30–34]. Most of the research in this area has focused on nurses, with little evidence available regarding the experiences of other disciplines in oncology. A 2013 secondary analysis of data from over 4,000 oncology nurses from 282 hospitals in three American states found 37% of nurses reported emotional exhaustion, 24% reported job dissatisfaction, 13% were likely to leave their job in the next year, and 18% reported poor or fair quality of care [30]. Although oncology nurses in that study reported more positive work and care delivery experiences compared to their medical-surgical counterparts, approximately one third perceived their working conditions as unfavorable. A more recent study of 200 RNs practicing in oncology settings in Sweden found nurses’ ability to deliver quality, safe patient care was compromised due to workplace conditions [33]. In a study of 18,719 physicians, approximately one third (~ 34%) reported higher than average rates of burnout, yet oncology physicians had lower than average rates of intent to leave compared to physicians in other specialties [34]. What remains unknown are the work and care delivery experiences of both nurses and other staff/clinicians at BC Cancer as they adapt to the new model of TBC.

## Methods

### Data collection

To evaluate the province-wide implementation of TBC across BC Cancer’s six regional Cancer Centres, a series of five longitudinal online surveys is planned to be administered over a two-year period. Eligible survey respondents include BC Cancer staff and clinicians working in the new TBC model at the time of each survey deployment. Surveys are administered using the Qualtrics online survey platform, with email invitations distributed to eligible respondents through organizational mailing lists.

Data were collected in the first of the five prospective surveys from May to June of 2023 during the early phase of TBC implementation. Pilot testing of the survey instrument prior to timepoint one was deferred due to time and resource constraints. Email invitations were sent to 299 eligible participants. Participation was encouraged through a multi-pronged approach, including organizational internal communications, email reminders, and a raffle draw incentive to win one of five $100 prepaid Visa cards per survey timepoint. Participants were informed about the confidentiality of their responses, particularly the limitations of data accessibility for organizational leadership (i.e., only aggregated findings would be shared), and the voluntary nature of survey participation. Harmonized ethics approval was granted by the University Behavioral Research Ethics Board and the BC Cancer Research Ethics Board (H22-02663). Given the bounded nature of our sample population—in that there was only a maximum of 299 team-based care staff eligible for the study—sample size requirements for our regression analyses were estimated based on general rule of thumb guidelines with an absolute minimum of 100 cases [35].

### Measures

The survey included questions on respondent demographics, including age as a continuous variable, and gender as a categorical variable with a write-in option. Other demographic variables included educational attainment, professional designation, employment status, and specialty area of practice. Respondents were asked about their overall experiences and perceptions across several key domains. This included subscales from the Guarding Minds at Work (GMW) framework [36] that assessed various aspects of respondents’ psychological health and safety in the workplace. The survey also included measures related to intent to leave their current job, quality of care delivered, patient safety, and likelihood of recommending their workplace from RN4CAST [37]. Specific survey items and response options used to assess work and care delivery experiences are detailed in Table 1. The survey also included a series of researcher-developed or researcher-adapted questions to capture perceptions of work and care delivery experiences related to the implementation of TBC. These survey questions and response options (Table 2) addressed four key areas: team effectiveness (researcher-developed), team composition (researcher-developed), team consistency (researcher-developed), and individual scope of practice (researcher-adapted) [23, 24] within the TBC framework. Respondents were also given the option to elaborate on their ratings of perceived team effectiveness and scope of practice by providing open-ended text responses. The survey took approximately 20–25 min to complete.

**Table 1:** Survey measures and items on perceptions of work and care delivery experiences

Construct	Survey Content	Response Options
Workplace Psychological Health and Safety	*Guarding Minds at Work* (4 select domains) **Organizational Culture (** ***5 items*** **)** - Difficult situations at work are addressed effectively. - I feel that I am part of a community at work. - Employees and management trust one another. - My workplace is inclusive of persons with diverse backgrounds and points of view. - Organizational values are demonstrated at all levels. **Engagement (** ***6 items*** **)** - I enjoy my work. - I am willing to give extra effort at work if needed. - My work is an important part of who I am. - I am committed to the success of my organization. - I am proud of the work I do. - I am committed to the success of my team. **Involvement and Influence (** ***5 items*** **)** - I am able to talk to my immediate supervisor about how I do my work. - I have some control over how I organize my work. - My opinions and suggestions are considered at work. - I am informed of important changes that may impact how my work is done. - My employer encourages input from all staff on important issues related to their work. **Workload Management (** ***5 items*** **)** - The amount of work I am expected to do is reasonable for my position. - I can talk to my supervisor about the amount of work I have to do. - I have the equipment and resources needed to do my job well. - My work is free from unnecessary interruptions and disruptions. - I have control over prioritizing tasks and responsibilities when facing multiple demands at work.	*Likert-type scale* 1. Strongly disagree 2. Somewhat disagree 3. Somewhat agree 4. Strongly agree
Intent to Leave	How likely are you to leave your job over the next year?	*Likert-type scale* 1. Very unlikely 2. Unlikely 3. Likely 4. Very likely
Quality of Care Delivered	*General* In general, how would you describe the quality of care you delivered to patients in your primary workplace? *Last shift* On your last shift, how would you describe the quality of care you delivered to patients in your primary workplace?	*Likert-type scale* 1. Poor 2. Fair 3. Good 4. Excellent
Patient Safety Grade	Please give your primary workplace an overall grade on patient safety.	*Likert-type scale* 1. Failing 2. Poor 3. Acceptable 4. Very good 5. Excellent
Likelihood of Recommending Workplace	*As a place for care* Would you recommend your primary workplace to your friends and family if they needed care? *As a place to work* Would you recommend your primary workplace to a colleague as a good place to work?	*Likert-type scale* 1. Definitely no 2. Probably no 3. Probably yes 4. Definitely yes

### Analysis

Quantitative data were analyzed using descriptive statistics and ordinal logistic regression (conducted using R and SPSS Version 29). Qualitative data analysis was guided by the applied methodology of interpretive description, wherein we employed analytic techniques (i.e., constant comparison, conceptual content analysis, and iterative pattern recognition) to illuminate applied practice insights within and across participants’ open-text responses [38].

For regression analyses, the Likert-type rating for *team effectiveness* was used as the ordinal outcome. For predictors, *team composition*, *team consistency*,* proportion of shifts practiced beyond scope*, and *proportion of shifts practiced below scope* were initially entered as continuous variables. Preliminary bivariate correlational analyses were conducted between the proposed regression variables to assess for multicollinearity and suitability for inclusion. The *team consistency* variable was measured using a 5-point Likert-type scale, which was then treated as a continuous variable in the regression analysis. To conserve power, *team composition* was removed from the final regression model, as analyses indicated that the 18 dichotomous variables included within the select-all-that-apply item were not independently correlated with team effectiveness. The frequency proportions for shifts *beyond*,* within*, and *below* scope were required to add up to 100% for each respondent. As a result, the proportions of shifts *within scope* were not entered as a separate predictor variable to avoid redundancy. The demographic variables of *age* and *gender* were entered as controls. The ‘prefer to describe’ level for *gender* was considered missing data for the regression model, due to convergence issues arising from low subgroup size (*n* = 3).

**Table 2:** Team-based care survey items, researcher-developed

Variable	Survey Item	Response Options	
Team Effectiveness	On average, how would you rate the overall effectiveness of your care team (inter- and intra-professional) on your work unit?	*Likert-type scale* 1. Very ineffective 2. Somewhat ineffective 3. Somewhat effective 4. Very effective	
	*Follow-up question*: What would you describe as the contributing factors to the effectiveness/ ineffectiveness of your care team?	*Open-ended long-form text response.*	
Team Composition	Who is part of your multidisciplinary team? (Select all that apply.)	*Select all that apply.*	
		- Medical Oncologist - Radiation Oncologist - General Practitioner in Oncology - Nurse Practitioner - Registered Nurse - Licensed Practical Nurse - Patient Care Aide - Unit Clerk - Pharmacist - Dietitian	- Indigenous Patient Navigator - Registered Social Worker - Registered Clinical Counselor - Speech Language Pathologist - Radiation Therapist - Physical Therapist - Occupational Therapist - Other (please specify)
Team Consistency	On average, how frequently do you work with the same individuals within your multidisciplinary team?	*Likert-type scale* 1. Never or rarely 2. About 25% of the shifts worked 3. About 50% of the shifts worked 4. About 75% of the shifts worked 5. Always	
Scope of Practice	Please think of the shifts you have worked in your current job over the last month, and consider whether you practiced beyond, within, or below your full/legal scope of practice in those shifts. What proportion of these shifts did you practice…	*Proportion sliders*,* each ranging from ‘None of the shifts worked (0%)’ to ‘All of the shifts worked (100%)’. The three sliders must add up to 100%.* - Beyond your scope of practice? - Within your scope of practice? - Below your scope of practice?	
	*Follow-up question*: In your opinion, what factors contribute to you practicing beyond/below your full/legal scope of practice?	*Open-ended long-form text response.*	

## Results

A summary of demographic information collected in the survey is shown in Table 3. A total of 120 complete or near-complete responses were received, yielding a response rate of 40%. The mean age of respondents was 41 years, and mostly consisted of women (*n* = 89; 75%), those working in direct patient care (*n* = 91; 77%), and those working full-time (*n* = 106; 90%). The top respondents were registered nurses (*n* = 39, 33%), physicians (*n* = 30; 26%), and unit clerks (*n* = 22; 19%). Approximately two-thirds of respondents worked in a systemic therapy program (*n* = 73), while the remaining one-third worked in a radiation therapy program (*n* = 38).

**Table 3:** Descriptive statistics and frequencies for demographic variables

Continuous Variable	*N*	Mean	SD	Min	Max
Age	116	41.03	11.03	22	72
**Categorical Variable**	**Level**			**Freq**	**Pct**
Designation (*N* = 117)	Registered Nurse			39	33.3
	Physician			30	25.6
	Unit Clerk			22	18.8
	Pharmacist			9	7.7
	Nurse Practitioner			7	6
	Licensed Practical Nurse			4	3.4
	*Other*			4	3.4
	Patient Care Aide			1	0.9
	Indigenous Patient Navigator			1	0.9
Education (*N* = 118)	Undergraduate degree			44	37.3
	Other Doctorate (MD, PharmD, DNP, etc.)			32	27.1
	Diploma/Certificate			30	25.4
	Master’s degree			10	8.5
	Doctor of Philosophy (PhD)			2	1.7
Gender (*N* = 118)	Woman			89	75.4
	Man			26	22
	*Prefer to describe*			3	2.5
Program (*N* = 118)	Systemic Therapy			74	62.7
	Radiation Therapy			38	32.2
	*Other*			6	5.1
Role (*N* = 118)	Direct patient care			91	77.1
	Clerical/Medical secretary			22	18.6
	Leadership/management			3	2.5
	*Other*			2	1.7
Status (*N* = 118)	Full-time			106	89.8
	Part-time			10	8.5
	Casual			2	1.7

### Descriptive statistics and qualitative insights

#### Workplace factors

A descriptive summary of variables on perceptions of work and care experiences is shown in Table 4. The GMW survey domain scores were calculated using the average scores of all items within each domain, with higher scores (maximum value = 4) representing more positive perceptions and lower scores (minimum value = 1) representing less negative perceptions. Ordered from most-positively to least-positively scored: *Engagement* had an average score of 3.51; *Involvement and Influence* had an average score of 2.86; *Organizational Culture* had an average score of 2.83; and *Workload Management* had an average score of 2.57.

#### Intent to leave

In this baseline assessment of TBC staff and clinicians, 74% of respondents reported they were ‘unlikely’ or ‘very unlikely’ to leave their job within the next year. Through open-ended text responses, participants highlighted how [1] a positive culture of reciprocal appreciation and mutual support (e.g., team cohesion, collaboration, feeling respected and valued within the team, fair treatment of everyone), and [2] the opportunity to provide more comprehensive person-centered care within TBC contributed to their intention to remain in their current role. As one participant shared:

*The feeling of community and being a part of the team goes a long way. It feels very rewarding to work alongside the physicians and NPs to give the client the care and attention I was unable to while in a different role in the centre. It has given me a different and more positive overall perspective of cancer care in general. [TBC RN]*

Participants cited several additional factors for remaining in their current role that were not attributable to TBC specifically, but nevertheless were viewed as important when determining intent to leave. These factors include: a passion for cancer care; opportunities for career advancement and further education; and a dependable, daytime-only clinical workweek (e.g., 8-hour shifts, Monday through Friday) with fewer disruptions and unpredictability than what is typically associated with shift-based schedules. As described by one participant:

*I really enjoy working in oncology because I find it fascinating and I get along with my colleagues. Working in team-based care and just getting out of the usual rotation has been a welcome change. Even though there will always be challenges in healthcare*,* I find what we do makes a difference in people’s lives and that is so meaningful to me. [TBC Pharmacist]*

When asked about strategies that would change their decision to leave their job, suggestions included: reducing the disproportionate workload faced by TBC team members due, in part, to staffing shortages and system inefficiencies (e.g., clinic workflows, appointment bookings); offering flexible work scheduling (e.g., a 4-day workweek); optimizing scope of practice; and minimizing the heavy administrative burden placed on healthcare providers.

*Time needs to be allocated for each task*,* and sometimes*,* in the amount of time given*,* not everything can be done. In order to delegate the tasks to other members of the team*,* there needs to be “other members” available (within the same scope and role) to delegate to.* [*TBC RN*]

Regarding staffing challenges, one person highlighted the difficulty of attracting specialized healthcare professionals to regions of the province with exceptionally high costs of living:

*Funding is much appreciated but obviously it will take years for the organization to recruit oncologists and other support staff.* [*TBC Medical Oncologist*]

Other respondents shared a range of concerns and challenges related to their feelings about potentially leaving their current role. One oncologist described experiencing moral distress due to lengthy patient wait times, which contributed to feelings of being unable to provide safe, effective care—factors that weighed into their considerations about leaving their role. Another respondent cited the challenge of keeping up with treatment advancements, and the need to address disrespectful behaviors in the workplace such as “bullying and horizontal violence.”

#### Quality and safety outcomes

Regarding staff and clinicians’ perceptions of quality of patient care, 93% of respondents rated their general care provision as ‘good’ or ‘excellent,’ and 95% held the same positive views specifically for their last shift. Patient safety grades were perceived positively, with 59.6% rating it as ‘very good’ or ‘excellent’ and 34% rating it as ‘acceptable.’ The likelihood of respondents recommending their workplace to friends and family as a place to receive care was high, with 87% indicating they would ‘probably’ or ‘definitely’ recommend it. Comparatively, 67% were ‘probably’ or ‘definitely’ likely to recommend their workplace to colleagues as a place to work.

**Table 4:** Descriptive statistics and frequencies for work and care experience variables

Continuous Variable	*N*	Mean	SD	Min	Max
*Workplace Psychological Health and Safety (GMW)*,* Domain Mean Score*					
Organizational Culture	115	2.83	0.66	1	4
Engagement	116	3.51	0.47	2.17	4
Involvement and Influence	114	2.86	0.71	1	4
Workload Management	116	2.57	0.76	1	4
**Categorical Variable**	**Level**			**Freq**	**Pct**
Intent to Leave (*N* = 110)	Very unlikely			39	35.5
	Unlikely			42	38.2
	Likely			23	20.9
	Very likely			6	5.5
Quality of Care, General (*N* = 107)	Poor			0	0
	Fair			8	7.5
	Good			53	49.5
	Excellent			46	43
Quality of Care, Last Shift (*N* = 107)	Poor			0	0
	Fair			5	4.7
	Good			51	47.7
	Excellent			51	47.7
Patient Safety Grade (*N* = 109)	Failing			0	0
	Poor			7	6.4
	Acceptable			37	33.9
	Very good			41	37.6
	Excellent			24	22
Likelihood of Recommending Workplace for Care (*N* = 108)	Definitely no			3	2.8
	Probably no			11	10.2
	Probably yes			46	42.6
	Definitely yes			48	44.4
Likelihood of Recommending Workplace for Work (*N* = 108)	Definitely no			12	11.1
	Probably no			24	22.2
	Probably yes			37	34.3
	Definitely yes			35	32.4

#### Team effectiveness, composition, and consistency

A descriptive summary of variables on team effectiveness, team composition, team consistency, and scope of practice is shown in Table 5. Team effectiveness ratings were generally positive, with 89% reporting a rating of ‘somewhat’ or ‘very’ effective. Team effectiveness was influenced by team composition, team consistency, and scope of practice.

When asked to identify members of their typical multidisciplinary team composition, 89% of respondents indicated they work with RNs, 82% work with unit clerks, and 78% work with medical oncologists as part of their typical team. A complete description of team composition is included in Table 5. Respondents described how inconsistencies in team composition created gaps that compromised a team’s ability to deliver comprehensive, patient-centered care. For instance, when essential roles such as clerical staff and LPNs were vacant due to sick calls or staff turnover, direct care staff spent a disproportionate amount of time on administrative tasks (i.e., printing patient materials, managing prescriptions, facilitating information flow) which detracted them from their core responsibilities (i.e., providing direct patient care).

The mean team consistency rating was 3.95, which corresponds to an average response of ‘75% of shifts worked’ with the same team members. In open-ended responses, participants identified that consistency of team members was critical for establishing workflows that enabled effective team functioning:

*When the nurses are pulled to other jobs/role*,* they are not available to do TBC related work*. [*TBC RN*]

Working consistently with the same team members and the development of interpersonal relationships was highlighted as a factor contributing to team effectiveness, as it led to the creation of a safe space where questions could be asked and preferences could be shared.

*It always helps to get to know each other better as humans rather than just as our professions. Being comfortable with each other definitely helps us work better as a team – I think everyone feels they can speak freely and ask questions without any fear of judgement*. [*TBC Pharmacist*]

Furthermore, team consistency and familiarity with individual clinicians’ treatment preferences (e.g., an oncologist’s preferred medication regime) were identified as factors contributing to both effective team functioning and enhanced operational efficiency.

*We know each other’s work preferences*, e.g.,* which antiemetics the oncologists like to prescribe*,* which [aromatase inhibitor] they prefer*,* how many days of filgrastim they like to start with etc. That way*,* either the pharmacist or RN can prepare prescriptions ahead of time and the oncologist can just sign off*,* making the process more efficient*. [*TBC Pharmacist*]

The physical environment was identified as a factor that can either facilitate or hinder communication and teamwork among staff:

*Working within the same space allows for access to your medical oncologist for questions*,* reports*,* ongoing plans of care*,* follow ups with [patient phone calls] and clinic issues.* [*TBC RN*]

*Space – if there was enough space*,* we can easily find each other to communicate. Currently we are physically separated* [*TBC RN*].

Another participant described a scenario where physical proximity posed challenges in how effective and efficiently a team worked together:

*I run clinic on my own with a [radiation oncologist] in a location separated from my partner who is running a clinic in a different area with another [radiation oncologist]*,* but we are all in the same team. . When I need help*,* I have to ask my other team member from a different location to help me. . having different locations does not help me work efficiently.* [*TBC RN*]

#### Scope of practice

Respondents reported that over the last month, on average, they practiced ‘below’ their scope of practice for 16% of shifts, ‘within’ their scope for 71% of shifts, and ‘beyond’ their scope for 13% of shifts. The majority of the participants described insufficient human resources as a contributing factor leading to them to practice below or beyond their scope. Specifically, participants highlighted how insufficient health human resources (i.e., across disciplines and across sectors) often led them to engage with activities below or beyond their roles and responsibilities.

“*Patients have no family doctors. I am often doing things that would usually fall in the scope of family docs. Numerous examples. Prescription refills for statins*,* treating HSV in patients not on chemotherapy*,* making referrals for cholazions of the eye in testicular cancer patients*.” [*TBC Medical Oncologist*].

“*Situations where more appropriate/qualified individuals aren’t available or present to address various issues*.” [*TBC NP*].

Participants acknowledged that insufficient human resources was secondary to high turnover rates.

“*High turnover rates especially in clerical and secretarial positions. No continuity of personnel*,* so physicians are left to deal with many clerical*,* scheduling and non-medical decision making issues*.” [*TBC Medical Oncologist*].

Having to practice below or beyond one’s own scope of practice was often perceived as increased workload:

“*Let me be a doctor. Not a clerk or nurse. Reduce my workload*.” [*TBC Medical Oncologist*].

*[There is a] lack of communication between team members regarding full scope. Other nurses [do not] want to*,* or [are not] able to practice at full scope. So if I practice at full scope*,* then I am unable to hand off to them.* [*TBC RN*]

As team composition grows, there is a need to clarify interdisciplinary expectations of what constitutes each team member’s unique scope of practice.

### Ordinal regression analysis

The variables included in the ordinal regression model included TBC-specific variables (i.e., team consistency, team effectiveness, and scope of practice) and demographic variables (i.e., age and gender). Of the 120 complete or near-complete survey responses, 27 had missing data for at least one of the included variables; after listwise deletion, the final regression sample size was 93 observations.

**Table 5:** Frequencies and descriptive statistics for proposed regression variables

Continuous Variable	*N*	Mean	SD	Min	Max
Age	116	41.03	11.03	22	72
Team Consistency^1^	105	3.95	1.00	1	5
Proportion of Shifts Below Scope of Practice	110	16.26	22.39	0	100
Proportion of Shifts Beyond Scope of Practice	110	13.01	21.94	0	96
**Categorical Variable**	**Level**			**Freq**	**Pct**
Gender (*N* = 115)	Woman			89	77.4
	Man			26	22.6
Team Effectiveness (*N* = 104)	Very ineffective			2	1.9
	Somewhat ineffective			9	8.7
	Somewhat effective			52	50
	Very effective			41	39.4
**Variable Set**	**Discipline (Binary Variable)**			**Freq** **(** ***Selected*** **)**	**Pct**
Team Composition^2^ (*N* = 105)	Registered Nurse			93	88.6
	Unit Clerk			86	81.9
	Medical Oncologist			82	78.1
	Nurse Practitioner			63	60
	Pharmacist			62	59
	General Practitioner in Oncology			58	55.2
	Licensed Practical Nurse			52	49.5
	Radiation Oncologist			52	49.5
	Patient Care Aide			46	43.8
	Dietitian			37	35.2
	Radiation Therapist			26	24.8
	Registered Social Worker			26	24.8
	Registered Clinical Counselor			25	23.8
	Indigenous Patient Navigator			24	22.9
	Speech Language Pathologist			15	14.3
	Physical Therapist			8	7.6
	Occupational Therapist			5	4.8
	Other (please specify)			1	1

^1^ Team consistency was collected as a 5-point Likert-type scale but was entered into the regression model as a continuous variable, with a score of 1= ‘Never or rarely’ and 5= ‘Always’

^2^ To conserve power, team composition was not included in the final regression model

The results of the ordinal logistic regression analysis are presented in Table 6. The SPSS Test of Parallel Lines was used to test the assumption of proportional odds, which was indicated to be met (*p* = .512). Multicollinearity diagnostics also showed that variance inflation factor values were below 10.

**Table 6:** Associations between ratings of team effectiveness, team consistency, and scope of practice

Variable		B	SE	*p*	OR (95% CI)
*Demographic Controls*					
Age		-0.036	0.020	0.068	0.96 (0.93, 1.00)
Gender	Woman *(reference category)*	-	-	-	-
	Man	0.533	0.514	0.300	1.71 (0.63, 4.79)
*Team-based Care*					
Team consistency		0.563	0.244	**0.021**	**1.76 (1.10**,** 2.88)**
Below scope		-0.056	0.014	**< 0.001**	**0.94 (0.92**,** 0.97)**
Beyond scope		-0.017	0.011	0.100	0.98 (0.96, 1.00)

Note: *p* <.05 indicates statistical significance

Team consistency and the proportion of shifts where respondents reported practicing below scope were both significant predictors of team effectiveness ratings. Specifically, higher ratings of team consistency were associated with higher ratings of team effectiveness. Each one-point increase along the five-point Likert scale for team consistency (e.g., *About 50% of the shifts worked* (3) to *About 75% of the shifts worked* (4)) was associated with 1.7 times the odds of a higher rating of team effectiveness (OR = 1.76; 95% Confidence Interval [CI]: 1.10–2.88). Higher proportions of shifts below scope were associated with lower ratings of team effectiveness. Each one-percent increase in the proportion of shifts practiced below full scope of practice within the last month was associated with 0.94 times the odds of a higher rating of team effectiveness (OR = 0.94; 95% CI: 0.92–0.97); or approximately 1.06 times the odds of a lower effectiveness rating (1/0.94). However, the proportion of shifts beyond scope was not significantly associated with ratings of team effectiveness.

## Discussion

This study offers a critical early-phase evaluation of TBC at BC Cancer, the publicly funded cancer control program for the westernmost Canadian province of BC. The concept of staff and clinicians working collaboratively in multidisciplinary teams is increasingly recognized as a necessity in complex clinical contexts like cancer care, where patients often require input from a variety of specialists across the care continuum [3, 39]. However, cancer care is often episodic, dynamic, and influenced by several factors including role clarity, communication, leadership, and organizational context [40]. Healthcare organizations must determine how best to support these teams and establish the necessary structures and safeguards in place to enable them to function effectively and cohesively. Optimizing TBC requires a nuanced understanding of what is working well and what challenges teams face. In examining the key dimensions of TBC, we can understand how well teams are functioning and identify areas for improvement.

Research from diverse clinical settings has described the role of team composition on the effectiveness of multidisciplinary teams. The extant literature underscores the importance of multidisciplinary approaches to care for improving quality of care and patient outcomes, which aligns with our findings [4, 5]. Our small sample size did not allow for the inclusion of the team composition variable in our regression analysis. We provide descriptive findings that offer insights about team composition, which may have implications for the operationalization of TBC. For instance, 89% of survey respondents indicated consistent collaboration with RNs; this not only highlights nursing’s pivotal role in team-based oncology care, but also gives justification for allocating proportional resources (e.g., practice supports, opportunities for continuing professional development, etc.) towards oncology nurses in this context. Future analyses in this longitudinal study will prioritize understanding how different permutations and combinations of team composition impact respondents’ perceptions of team effectiveness.

As TBC continues to evolve in the cancer sector, it is important to explore how integrating additional healthcare disciplines, such as clinical pharmacists and radiation therapists, could further optimize team dynamics and functionality. However, a comprehensive integration plan is required to effectively integrate new roles into team-based cancer care. If healthcare organizations fail to establish a clear roadmap for integrating additional clinicians, role confusion could arise and potentially exacerbate existing challenges within a TBC framework [8]. In alignment with conclusions drawn from a recent scoping review of team-based diabetes care in primary care settings, our results underscore the importance of fostering a strong sense of role clarity to ensure that all members of a multidisciplinary team are well-integrated and understand the unique contributions of each team member [17].

Our results also highlight the fundamental importance of team consistency. In our study, respondents who indicated that they frequently work with the same team members reported (a) higher perceptions of team effectiveness, and (b) greater continuity of care—a critical aspect for the optimal management of chronic diseases like cancer [3, 41]. Consistent with previous studies, our research identified staffing shortages and high turnover rates as significant impediments to maintaining team consistency [42, 43]. Our findings also reinforce the notion that TBC requires consideration not only for *who* is on a team, but also *where* and *how* they collaborate [9, 17]. Optimizing physical spaces for collaboration—in concert with other activities designed to improve collaboration, team consistency, and role clarity—may be positively associated with team consistency and effectiveness.

Delineating and aligning roles, responsibilities, and scopes of practice across different healthcare disciplines is of paramount importance for team-based oncology care. Importantly, we found a significant negative association between an increased proportion of the number of shifts where respondents were underutilized and perceptions of team effectiveness. Addressing this issue requires a multifaceted approach that may include adequate staffing; comprehensive training; knowledge mobilization about scopes of practice among various disciplines; removing the structural barriers that inhibit staff and clinicians from fully utilizing the spectrum of their professional skills; and addressing the structural determinants of the health human resource crisis [9, 20–25, 44].

The intention to leave among staff—which respondents noted was influenced by disproportionate workloads and moral distress associated with system constraints—highlights the broader implications of psychological health and safety among the oncology workforce. Worth noting, the aggregated results from this first timepoint of our prospective cross-sectional study reflect recent estimates of the psychological health and safety of BC’s nursing workforce [45, 46]. It is imperative that TBC is understood not only as an intervention to improve quality and effectiveness of cancer care, but also as a structural intervention that has the potential to improve the psychological health and safety outcomes among workers in this essential sector of our healthcare system. Thus, clinical and operational leaders must regularly and proactively assess the working conditions of oncology staff and clinicians for factors that affect the mental health and well-being of multidisciplinary teams.

In this study we examined respondents’ perspectives about their other workplace experiences and also quality and safety of patient care. Our findings supported strong job satisfaction, a high standard of patient care, and a sense of endorsing the workplace as both (1) a high-quality place to receive care, and (2) a high-quality place of employment. Our research team will use our longitudinal survey data to prospectively evaluate the impact of TBC on quality and safety indicators over time. The results of this study on the implementation of TBC at BC Cancer in BC, Canada should be considered in the context of their strengths and limitations. The prospective cross-sectional survey methodology provides an effective mechanism to evaluate the early-phase effects of TBC, capturing a range of measures on team effectiveness, perceptions of care including quality and safety outcomes, and scope of practice among staff and clinicians from across the province. However, generalizability of the study findings is limited due to over-representation of RNs in the data and our singular provincial focus which potentially restricts the applicability of these findings to other clinicians and jurisdictions. Additionally, the reliance on data from a single time point restricts insights into the evolution of TBC over time and establishing cause and effect across TBC factors and outcomes. The sample (predominantly comprised of nurses, clerical staff, and physicians) may not fully represent the broader disciplines that are currently peripherally involved with TBC but have not yet been integrated as core members of the TBC model. While this study does not have quantitative data to support the impact of team composition, the qualitative data supports its importance for team effectiveness. Future research will evaluate this relationship quantitively using longitudinal data. There is a possible response bias from those more inclined to participate due to strong opinions, which could potentially skew the results. Furthermore, the survey may not have sufficiently captured crucial teamwork dynamics such as communication, coordination, and staff/clinician well-being which are important for a comprehensive assessment of the effectiveness of TBC.

As the implementation of TBC becomes more widespread, understanding the key drivers of effective teamwork and sustainability will be essential to optimizing care quality, patient experience and outcomes, and staff wellness. The findings from this longitudinal research study, for which there are four subsequent timepoints, will be taken up by operational and practice leaders to adapt the implementation and expansion of TBC at BC Cancer.

Future research by our team will include longitudinal assessment of these key domains of TBC, operationalized through the administration of four additional prospective surveys over a two-year period. This body of work will allow us to examine TBC’s development and its long-term impact on team effectiveness and patient care at BC Cancer. As subsequent phases of TBC are implemented across regional Cancer Centres, expanding the demographic and disciplinary diversity of survey respondents may enhance the representativeness of the findings. Expanding assessments of teamwork dynamics such as communication, coordination, and shared mental models could provide greater insights into the mechanisms that influence the effectiveness of TBC [47].

## Conclusions

Using cross-sectional survey data, we evaluated the early implementation of TBC in outpatient oncology settings in one Canadian province. Baseline descriptive statistical analysis revealed that cancer care clinicians and staff reported positive perceptions regarding both their workplace experience and the quality of care delivery. We found that team consistency and scope of practice were significant predictors of team effectiveness. Higher team consistency correlated with higher effectiveness ratings, whereas more frequent instances of practicing below full scope corresponded to lower effectiveness ratings. These preliminary findings demonstrate the importance of team consistency scope optimization as essential drivers of effective teamwork. The contextual factors that shape healthcare professionals’ perceptions of effectiveness (e.g., team composition, role clarity, workload, and physical proximity of team members in the clinical space) are important areas for future investigation. These insights highlight key areas that warrant focused attention by healthcare organizations to develop and implement tailored strategies that foster high-functioning multidisciplinary teams and improve the quality of cancer care services.

## Data Availability

The dataset analyzed during the current study are not publicly available due to ethics requirements but are available from the corresponding author on reasonable request and after obtaining appropriate ethics boards’ approval.

## Abbreviations

TBC: Team-based care
BC: British Columbia
NP: Nurse Practitioner
RN: Registered Nurse
LPN: Licensed Practical Nurse
PCA: Patient Care Aide
IPN: Indigenous Patient Navigator
GMW: Guarding Minds at Work evaluation tool for workplace psychological health and safety
